# Exploring Factors Affecting Crash Injury Severity with Consideration of Secondary Collisions in Freeway Tunnels

**DOI:** 10.3390/ijerph20043723

**Published:** 2023-02-20

**Authors:** Younshik Chung, Jong-Jin Kim

**Affiliations:** 1 Department of Urban Planning and Engineering, Yeungnam University, Gyeongsan 38541, Republic of Korea; 2 Legislation Office, Gyeongsangnam-do Provincial Council, Changwon 51139, Republic of Korea

**Keywords:** freeway tunnel, crash injury severity, indirect effect, structural equation modeling, secondary collisions

## Abstract

Although there have been several studies conducted exploring the factors affecting injury severity in tunnel crashes, most studies have focused on identifying factors that directly influence injury severity. In particular, variables related to crash characteristics and tunnel characteristics affect the injury severity, but the inconvenient driving environment in a tunnel space, characterized by narrow space and dark lighting, can affect crash characteristics such as secondary collisions, which in turn can affect the injury severity. Moreover, studies on secondary collisions in freeway tunnels are very limited. The objective of this study was to explore factors affecting injury severity with the consideration of secondary collisions in freeway tunnel crashes. To account for complex relationships between multiple exogenous variables and endogenous variables by considering the direct and indirect relationships between them, this study used a structural equation modeling with tunnel crash data obtained from Korean freeway tunnels from 2013 to 2017. Moreover, based on high-definition closed-circuit televisions installed every 250 m to monitor incidents in Korean freeway tunnels, this study utilized unique crash characteristics such as secondary collisions. As a result, we found that tunnel characteristics indirectly affected injury severity through crash characteristics. In addition, one variable regarding crashes involving drivers younger than 40 years old was associated with decreased injury severity. By contrast, ten variables exhibited a higher likelihood of severe injuries: crashes by male drivers, crashes by trucks, crashes in March, crashes under sunny weather conditions, crashes on dry surface conditions, crashes in interior zones, crashes in wider tunnels, crashes in longer tunnels, rear-end collisions, and secondary collisions with other vehicles.

## 1. Introduction

Roadway tunnels are generally used to overcome mountainous terrain, but crashes that occur in them have greater severity than those on open road sections [1,2,3,4,5,6,7]. This may be caused by temporary blindness due to the abrupt change in light conditions, distraction, and reduced concentration due to the monotonous tunnel interior, as well as the late medical response for injured people due to the narrow tunnel space. These tunnel characteristics can affect injury severity in crashes and crash characteristics, such as collision types and crash causal factors. Specifically, when considering the definition of a secondary collision, which implies a collision with two or more consecutive impacts regardless of the initial crash type, crashes in tunnels are more likely to be secondary collisions due to differences in the environment from open road sections, such as poor light conditions, narrow shoulders, and tunnel walls. Although secondary collisions contribute to severe injuries in vehicle crashes, their relationship with the severity of crash injuries has not been fully explored [8]. In particular, a few studies have been conducted on vehicle crashes [8,9,10,11,12,13], motorcycle crashes [14,15], and vehicle-to-pedestrian crashes [16] in a general roadway environment. Nevertheless, there has been no effort to identify the relationship between secondary collisions and the injury severity of a crash in a freeway tunnel environment.

In analyzing the injury severity in traffic crashes, most previous studies have used injury severity information scaled into three to five classes based on the most severe consequence of a crash, regardless of the number of vehicles or occupants involved. For example, the KABCO scale established by the Federal Highway Administration [17] is classified into five categories: fatal injury (K), incapacitating injury (A), non-incapacitating injury (B), possible injury (C), and property-damage-only (O). Due to the categorical or ordinal nature of these variables, the discrete outcome models such as multinomial logit/probit models and ordered logit/probit models are the top candidates for analyzing the injury severity of the crashes. However, such models may have a limitation due to endogenous interrelationships among injury severities in multi-vehicle crashes [18]. In addition, some independent variables may affect injury severity indirectly through one or more variables [19]. For example, driver and/or crash characteristics can directly affect injury severity. However, the tunnel characteristics can affect crash characteristics, and these crash characteristics can affect injury severity, meaning that the tunnel environment can indirectly affect injury severity.

Based on this background, the objective of this study was to explore factors affecting injury severity with the consideration of secondary collisions in freeway tunnel crashes. To account for complex relationships between multiple exogenous variables and endogenous variables by considering the direct and indirect relationships between them, this study used a structural equation modeling with tunnel crash data obtained from Korean freeway tunnels from 2013 to 2017. Specifically, to reduce the possibility of underreporting and inaccuracy of crash data and to include unique crash characteristics such as secondary collisions, this study used crash data reconstructed by high-definition closed-circuit television (CCTV) cameras installed at every 250 m to monitor incidents in Korean freeway tunnels.

## 2. Literature Review

### 2.1. Prior Studies on Injury Severity Analysis of Tunnel Crashes

Many studies have modeled the injury severity of crashes over the past few decades. Comparatively fewer studies, however, have modeled the injury severity of tunnel crashes because of the low portion of mountainous roadways or tunnel lengths in various countries. As shown in Table 1, most studies have been carried out in very mountainous nations, such as Norway, Italy, and mountainous regions in China, as well as other countries with river-crossing tunnels. Furthermore, descriptive statistics have generally been used to find the risk factors that affect the injury severity of tunnel crashes. On the other hand, recent studies have applied more complicated methods, such as ordinal models, decision trees, and random forest algorithms. Moreover, influencing factors that affect injury severity in tunnel crashes are tunnel locations in terms of tunnel zones, tunnel length, and width (or the number of lanes); average daily traffic and truck percentage; crash characteristics such as the involvement of heavy-duty trucks, and the number of involved vehicles; environmental characteristics including crash time, weather conditions, and road surface conditions; and driver characteristics such as gender, distraction, and age. Additionally, all studies except one in Singapore used police-recorded crash data, which may have underreporting issues and may not include detailed crash information such as secondary collisions.

### 2.2. Methodological Review for Injury Severity Analysis

Two types of methodological approaches are typically considered to analyze crash injury severity: statistical methods and machine learning methods. First, the statistical approaches that have been applied to model the severity of a crash injury can be classified generally as nominal models and ordinal models [28,29]. Researchers often select one of these models when considering crash data characteristics because each type has its benefits and limitations [29,30,31]. For example, ordinal models such as ordered logit/probit and generalized ordered models have been used widely to account for the ordinal nature of crash injury data. However, when crashes with property damage only (PDO) or minor injuries, such as pain and slight injury, are far more likely to be underreported, researchers suggest using nominal models rather than ordinal models to obtain correct parameter estimates [28,29,30]. On the other hand, nominal models such as multinomial logit/probit models do not preserve the ordinal nature of crash injury data. Consequently, they could result in a loss of efficiency in the estimated model [32]. Moreover, traditional multinomial logit/probit models suffer from constant parameter assumptions across all crashes or unobserved heterogeneity in the crash data [28,33,34]. Previous studies have discussed other benefits and disadvantages of these two types of models, e.g., [28,29,30,35,36,37].

In the analysis of injury severity, there have been various efforts to account for the heterogeneity in the crash data. They include, for example, random parameter models, e.g., [38,39,40], latent class clustering or latent segmentation models, e.g., [41,42,43], copula-based models, e.g., [34,44,45], Markov switching models [46,47], and SEM, e.g., [8,48,49]. Of these, SEM can effectively establish multiple relationships between or within endogenous and exogenous variables simultaneously and incorporate latent variables into the model to bridge the gaps between them [48,49,50]. Unlike conventional regression models, where there is a clear distinction between dependent and independent variables, such concepts in SEM only apply in relative terms because a dependent variable in one model equation can become an independent variable in other components of the SEM system [51,52].

Machine learning methods can be considered another methodological approach. These methods have recently been applied to overcome the limitations of statistical methods, such as predefined assumptions, multicollinearity, and unobserved heterogeneity, e.g., [53,54,55,56,57,58]. Moreover, machine learning methods have been reported to provide a powerful prediction and classification of injury severities [56]. On the other hand, they do not directly report the conceptual interpretation of the model, and sensitivity analysis is usually conducted using a complicated process [59].

### 2.3. Literature Review Summary

A review of the literature showed that relatively few studies (fewer than 20) have been conducted to explore factors affecting the injury severity of tunnel crashes over a couple of decades. In addition, most of these studies used police-recorded crash data or crash data recorded by roadway operation agencies, which are more likely to have underreporting issues. Moreover, it was found that the location of tunnel zones, the geometric characteristics of the tunnel—such as tunnel length, curve, grade, and the number of lanes (or tunnel width—the characteristics of crashes, driver characteristics, and environmental characteristics are factors directly affecting the injury severity. On the other hand, there were no studies that considered secondary collisions in roadway tunnels.

In addition, most studies have analyzed the injury severity based on one severity variable, such as the number of fatalities or injury severity levels. Although the injury severity can be considered from multiple perspectives simultaneously, such as the number of fatalities, number of injured, and the level of injury severity, no study has considered the injury severity of tunnel crashes from multiple perspectives simultaneously. Moreover, in modeling, some independent variables may affect the injury severity indirectly through one or more variables. In particular, these tunnel characteristics can directly affect the crash characteristics and the injury severity considering the inconvenient driving environment in tunnel space, such as a narrow space and dark lighting. On the other hand, the tunnel environment can influence the crash characteristics, directly affecting the injury severity. Hence, the tunnel characteristics can indirectly affect the injury severity. Despite this, no effort has been made to prove their relationship.

## 3. Data Preparation

### 3.1. Data Collection

This study combined two data sources from the Korea Expressway Cooperation (KEC): 1790 crashes in tunnels on 30 freeways in Korea from 2013 to 2017 and tunnel inventory data for the relevant freeways. On the other hand, crash data were basically recorded by the police in a pre-specified format and information type. The KEC then reconstructed the crash data using high-definition CCTVs in tunnels. Tunnels in Korean freeway systems are the dual-tube type, and CCTV cameras are installed every 250 m in tunnels. Each camera monitors incidents in a tunnel length of approximately 125 m because these cameras have various functions, such as zooming, panning, and tilting. Therefore, when an incident, such as a crash, occurs, the staff at the traffic management center of the KEC can remotely confirm the scene of the incident and summarize the situation. The tunnel crash data are supplemented with crash data reported by incident response teams (including the police). Therefore, since most minor crashes that can lead to underreporting are detected and reconstructed by these CCTVs, underreporting issues could be minimized.

The crash data include the following: the time; causal factors; characteristics, such as crash type (rear-end collision or others); secondary collisions; type of vehicle that caused the crash; location; severity; environmental conditions (such as weather and surface conditions); roadway characteristics (such as vertical and horizontal curvatures); and demographic characteristics (such as the age and gender of the driver who caused the crash). Lane changing is restricted in most sections of freeway tunnels in the Korean roadway system, so only two types of crashes were considered: rear-end collision, which is primarily between leading and following vehicles, and others, which are primarily between a vehicle and other objects, such as the tunnel facilities, debris in the tunnels, and falling objects. In this study, the variable “secondary collision” was defined as a crash with two or more impacts regardless of the initial crash type, and it is differentiated from a “secondary crash,” which is generally defined as a crash occurring within the spatiotemporal regions of an initial crash. This variable could be reconstructed based on the high-definition CCTV recordings. Because these impacts could be due to a single vehicle alone or multiple vehicles, this study classified secondary collisions into two types: single-vehicle and multi-vehicle.

### 3.2. Definition of Tunnel and Crash Data

The KEC initially categorized the severity of crash injuries into four-level ordinal scales: (1) property damage only (PDO), complaint of pain requiring hospitalization for less than five days, or first-aid treatment at the crash scene; (2) minor injury requiring hospitalization for three weeks or less; (3) incapacitating injury requiring hospitalization for more than three weeks; and (4) fatal injury. On the other hand, these two categories were combined into “killed and seriously injured (KSI)” due to the low frequency of incapacitating and fatal injuries.” Thus, the response variable was categorized into three-level ordinal scales based on the increasing degree of severity and coded as follows: 1 = PDO, 2 = minor injury, and 3 = KSI.

**Figure 1:**
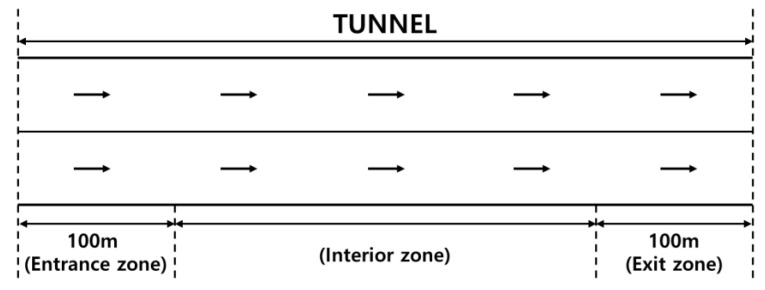
Sub-divided tunnel zones.

The proportions of KSI and minor injury crashes in the Korean freeway tunnels were 3.80% and 10.06%, whereas those in open sections were 3.52% and 6.40%, respectively. Therefore, this study confirmed that the risk of being killed or injured is higher in tunnels than on open roadway stretches. The tunnel inventory data include the length, width, and height of the tunnel and the number of lanes. As mentioned in previous studies, tunnel zones affect the severity of crash injuries. Most studies divided freeway tunnels into three sub-zones: the entrance, interior, and exit, as shown in Figure 1. Moreover, significant differences have been found in the injury severity at four different lengths of the entrance/exit zones: 100 m [2,23,25], 150 m [1,22], 250 m [5], and 300 m [3]. To select the appropriate length of the entrance/exit zone, the effects of different lengths on the injury severity were tested based on 50 m increments. An ordered probit model was applied to analyze the relationships between injury severity and incremented zone lengths, and 100 m was selected. Because data on crashes in tunnels were used, the zone in front of the tunnel openings was not defined. Each crash was associated with one of the divided tunnel zones.

**Figure 2:**
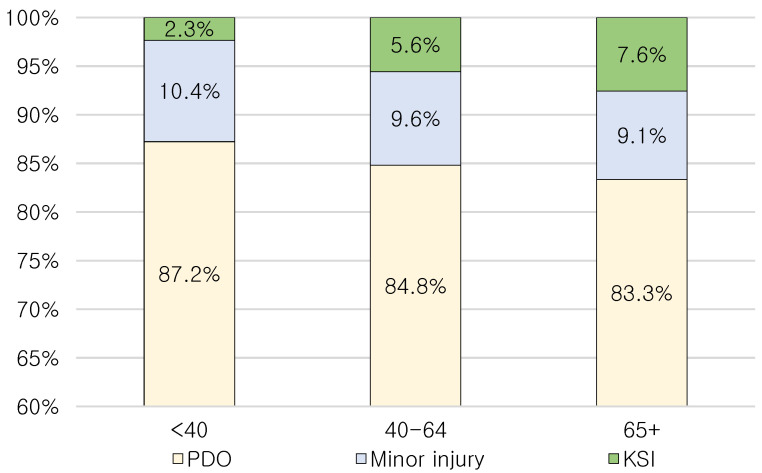
Crash proportion and frequency with respect to age classes.

In general, the age-related deterioration of mental and physical ability is associated with increased crash frequency and injury severity. Of various mental and physical abilities, the visual ability of drivers would be a critical factor in the tunnel environment, where there is a “black hole” at tunnel entrances and a “white hole” at tunnel exits. According to a report by the Mayo Clinic (www.mayoclinic.org: accessed on 1 February 2023), the gradual loss of the eyes’ ability to focus on nearby objects begins after the age of 40, and it is called presbyopia. To identify this effect, this study categorized the age-related variable into three groups: drivers younger than 40 years old (age_group_1), drivers aged 40 to 64 years old (age_group_2), and drivers aged 65 years and above (age_group_3). Figure 2 shows the crash proportions and frequencies with respect to the three age groups. As expected, the injury severity of tunnel crashes increased with older age groups. The other candidate variables shown in Table 2 were also defined based on a previous study and the data properties obtained.

## 4. Methodology

### 4.1. Structural Equation Modeling

This study used structural equation modeling (SEM) to identify the relationship between injury severity and its causal factors while considering indirect effects. SEM is a statistical method for testing and estimating causal relationships using a combination of statistical data and qualitative causal assumptions [60]. According to Chin [61], it specifically provides the researcher with the flexibility to (1) model relationships among multiple independent variables and dependent variables, (2) construct unobservable latent variables, (3) model errors in measurement for observed variables, and (4) statistically test a priori substantive/theoretical and measurement assumptions against empirical data (i.e., confirmatory analysis). Therefore, it is widely used in the social sciences, such as psychology, sociology, political science, and market research. Recently, it is also been widely applied in travel behavior research, e.g., [62,63,64,65] and traffic safety research, e.g., [19,48,49,50,66].

Although traditional statistical methods, such as multiple regression, path analysis, factor analysis, time series analysis, and analysis of covariance, may be viewed as special cases of SEM, SEM has been used as a more powerful alternative to them. It is generally viewed that the advantages of SEM compared to multivariate statistical methods include more flexible assumptions, particularly allowing interpretation even in the face of multicollinearity, the use of confirmatory factor analysis to reduce measurement error by having multiple indicators per latent variable, the attractiveness of SEM’s graphical modeling interface, the desirability of testing models overall rather than coefficients individually, the ability to test models with multiple dependents, the ability to test coefficients across multiple between-subjects groups, and the ability to handle difficult data, such as time series with autocorrelated error, non-normal data, and incomplete data [60,67].

### 4.2. Components of SEM

The general SEM model consists of two sub-models: the measurement and structural models. The measurement model specifies the relationships between the observed and unobserved latent variables. Therefore, this model provides the link between scores on the observed variables and the underlying constructs they are designed to measure (i.e., the unobserved latent variables). On the other hand, the structural model specifies the relationships among the unobserved latent variables. Accordingly, it specifies how particular latent variables directly or indirectly influence (i.e., cause) changes in the values of certain other latent variables in the model [68]. Since two types of latent variables (i.e., endogenous and exogenous latent variables) are considered in SEM, some researchers have described SEM as having three components [48,49]: (1) a measurement model for the endogenous variables, (2) a measurement model for the exogenous variables, and (3) a structural model.

Exogenous latent variables always act as independent variables and never have error terms. On the other hand, endogenous latent variables always act as dependent variables and always have error terms. Nevertheless, they can also act as independent variables impacting other endogenous variables. In the injury severity analysis of tunnel crashes, a tunnel factor related to tunnel width and length can be considered an exogenous latent variable, while injury severity factors, including the number of fatalities, number of injured, and injury severity, can act as endogenous latent variables.

**Figure 3:**
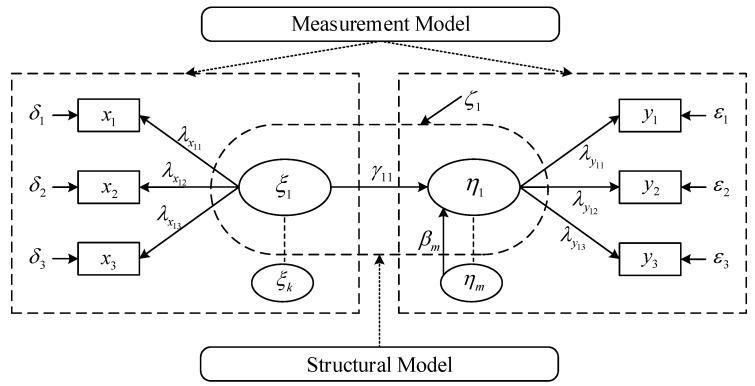
An example of SEM.

Figure 3 shows an example of a general SEM. In the figure, the rectangles and ellipses represent observed and unobserved latent variables, respectively, and the unenclosed variables signify error terms. Moreover, the arrow signifies the relationships between two variables, and the coefficient located in the middle of the arrow signifies the relationship between the connected two variables. Thus, the directional arrow implies one variable having a direct effect on another. Table 3 presents the SEM terms and notation in Figure 3.

### 4.3. Model Specification

Model specification occurs when one specifies which relationships are hypothesized to exist or not to exist among observed and unobserved latent variables, and it is generally considered the first step in SEM [69]. Thus, SEM requires specifying a structural model based on theory and expectation and identifying latent variables that can be measured by observed variables [19,30,70]. Therefore, the results from previous studies can be a basis for constructing model hypotheses. Moreover, based on the scope and rationale of the study, the conceptual model and hypotheses are substantially proposed.

### 4.4. Assessment of SEM

Assessing a model fit provides an overall perspective on how well the theoretical model can reproduce the observed data. There have been various criteria developed for assessing the overall goodness-of-fit of an SEM. According to a previous study by Chung and Kim [60], two popular methods can be considered to evaluate an SEM: (1) the $\chi^{2}-test$ statistic assessing the magnitude of discrepancy between the sample and fitted covariance matrices, and (2) fit indices quantifying the degree of fit along a continuum. Although only the $\chi^{2}-test$ statistic is available for inferential statistical evaluation of an SEM, it suffers from two types of limitations: (1) the assumption of multivariate normality, so that severe deviations from normality may result in model rejections even when properly specified, and (2) sample size issues, so that it nearly rejects the model in the case of large samples or that it lacks statistical power in the case of small samples.

As a result, most experts suggest that it should be used as a measure of fit rather than as a test statistic [71]. As representative fit indices using cutoff criteria, they have developed alternative indices such as descriptive goodness-of-fit measures, including the comparative fit index (CFI), goodness-of-fit index (GFI), and adjusted goodness-of-fit index (AGFI), and root mean square error of approximation (RMSEA). Although the acceptable limits for a well-fitting model may vary with study purpose, data properties, and sample size, Schermelleh-Engel, et al. [72,73] suggested desirable criteria for them, as shown in Table 4.

## 5. Injury Severity Analysis

### 5.1. Conceptual Model and Hypotheses

Unlike other social science studies using an SEM, this study utilizes already collected crash data. Therefore, the conceptual model and hypotheses are built on the relationship between the data collected, not the design of the study for data collection. In general, driver characteristics, environmental characteristics, roadway characteristics (or tunnel characteristics), and vehicle characteristics influence injury severity in crashes. Moreover, crash characteristics can also affect the injury severity in a crash. However, when considering the tunnel environment, the tunnel characteristics can also influence crash characteristics. That is, the tunnel characteristics may indirectly affect the injury severity. In addition, the injury severity can be considered with respect to injury severity itself, the number of injured, and the number of fatalities.

**Figure 4:**
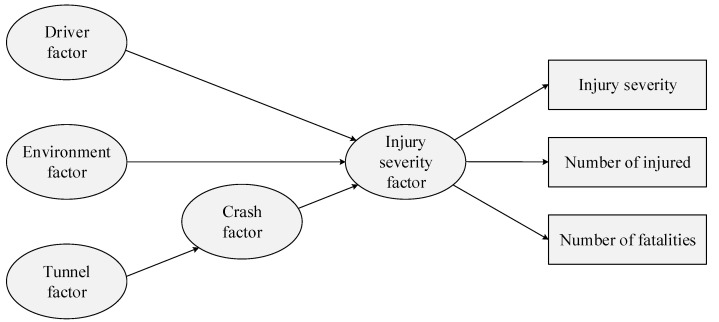
The conceptual model of injury severity in tunnel crashes.

In SEM, these characteristics related to observed variables can be considered latent variables [48]. Of these characteristics related to observed variables, vehicle characteristics can include vehicle type, weight, engine size, and vehicle age. However, only variables regarding vehicle type were available in this study. Thus, these variables were included in driver characteristics as used by Lee, Chung, and Son [48]. Based on the characteristics of crash and tunnel data as well as the results of the literature review, this study proposed a conceptual model, shown in Figure 4, and relational hypotheses as follows:

**H1:**  *Driver characteristics are interconnected with injury severity.*

**H2:**  *Environmental characteristics are interconnected with injury severity.*

**H3:**  *Tunnel characteristics are interconnected with injury severity through crash characteristics.*

### 5.2. SEM Results

**Figure 5:**
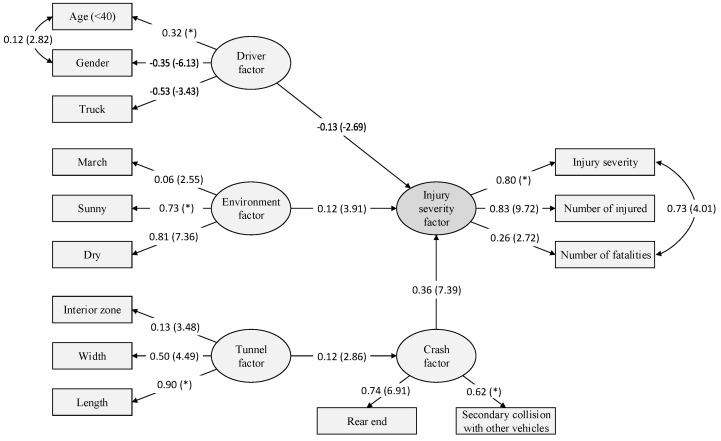
Final SEM for injury severity in freeway tunnel crashes.

When applying SEM, the estimation method should be selected. There have been several estimation procedures developed for SEM. They include maximum likelihood (ML), least squares (LS), unweighted LS, generalized LS, and asymptotic distribution free (ADF) or weighted LS [73]. The selection of the estimation method can be based on the distribution assumption and the size of the data. In the case of estimating SEM with non-normal data and very large samples (i.e., n≥500), researchers may choose ADF [69]. Since a significant portion of variable types in this study is categorical (or dummy), and the sample is larger than 500, SEM based on ADF estimation is conducted. Figure 5 presents the estimated SEM for injury severity in freeway tunnel crashes in a standardized form. In this figure, the values in the arrows are parameters estimated at the 95% significance level, and the values and asterisks in parentheses indicate the t-value and fixed parameters, respectively.

As shown in the figure, the test results of the proposed hypotheses were statistically significant at the 95% confidence level as follows:

**H1:**  *Driver characteristics are interconnected with injury severity (accepted).*

**H2:**  *Environmental characteristics are interconnected with injury severity (accepted).*

**H3:**  *Tunnel characteristics are interconnected with injury severity through crash characteristics (accepted).*

More specifically, all observed variables positively influence the injury severity, except the variable ‘Age (<40)’ that indicates crashes by drivers younger than 40 years old. In Figure 5, the variable ‘Age (<40) is positively related to the latent variable ‘Driver factor’, which is negatively associated with the latent variable ‘Injury severity factor’. Thus, crashes by drivers younger than 40 years old tend to decrease injury severity, the number of injured, and the number of fatalities in tunnel crashes. Other variables are similarly interpreted. The following section discusses the detailed interpretations of the relationships between endogenous and exogenous, latent and observed variables.

Table 5 summarizes the goodness-of-fit of the estimated SEM. The estimated model has an $\chi^{2}$ value of 221.302 with 68 degrees of freedom (df). Since the $\chi^{2}$ test is sensitive to sample size, it should not serve as the sole basis for evaluating model fit. Therefore, other fit indices such as RMSEA, CFI, GFI, and AGFI are reported additionally. Although the value of CFI is slightly less than the suggested acceptable fit ranges, other indices suggest that the model fits acceptably.

### 5.3. Interpretation of SEM Results

#### 5.3.1. Driver Factor

First, crashes by drivers younger than 40 years old were associated with decreased injury severity. This can be explained by presbyopia, which is the gradual loss of the eyes’ ability to focus on nearby objects, which begins after the age of 40 (www.mayoclinic.org: accessed on 1 February 2023). One of the symptoms is greater sensitivity to light and glare, resulting in temporary ocular blindness due to abrupt changes in the light conditions in tunnels. Another possible explanation can be interpreted as resulting from the greater physical flexibility of younger people. Their improved muscle tone could also allow them to adjust when colliding with an immediate skeletomuscular response to the event. The greater elasticity of their bone cartilage and muscle could reduce energy transmission to the bone [16]. Conventionally, older age groups in traffic accident analysis have been defined as those aged 65 years or older. Although more detailed research is required for the generalization, injury severities in tunnel crashes according to driver age group may be distinct at the age younger than 40 years old (or the age of 40 and above). This implies that the eyes’ ability in a low-illumination environment, such as a tunnel, differs from that in open roadways. Thus, if the driving ability of older drivers is considered for designing a tunnel environment, one possible classification criterion for the age group might not be chronological age but the eye’s ability to adapt to various illumination environments.

In addition, crashes by male drivers were found to increase injury severity, which is consistent with previous studies [23,25]. This result can be explained by the higher risk-taking driving behavior of male drivers. That is, since male drivers tend to drive their vehicles with higher risk behaviors such as fatigued driving, speeding, and aggressive driving [74,75,76], they could result in higher injury severity in tunnel crashes. Moreover, crashes with heavy vehicles in tunnels are more likely to involve severe injuries [23]. Other studies have reported similar results, e.g., [77,78,79]. This result can be interpreted based on the mass of heavy vehicles or kinetic energy transfer during a collision [80]. Crashes with a truck could result in severe injuries because a truck has a high mass.

#### 5.3.2. Environment Factor

As for the ‘environment factor,’ three variables led to a higher likelihood of severe injuries: crashes during March, crashes under sunny weather conditions, and crashes on dry road surface conditions. The first result regarding crash occurrence month could be interpreted in two ways. The first is based on topographic and seasonal effects. Because 63.0% of South Korean territory is covered by mountains [81], the cumulative length of tunnels comprises approximately 11.0% of the Korean freeway systems (451 km out of 4113 km), and 264 of the 1059 tunnels (24.9%) are more than one kilometer long (www.ex.co.kr: accessed on 1 February 2023). Moreover, the Korea Meteorological Administration (KMA) defines spring as beginning on 9 March and ending on 21 May. Therefore, most Koreans recognize March as the spring season, and they tend to drive less carefully in tunnels than in winter. Additionally, excluding the southern islands with no freeways, the average temperature and average minimum temperature in March on the Korean peninsula over the past 10 years were 5.3 and −0.1 °C, respectively (www.kma.go.kr: accessed on 1 February 2023). The average temperature and weather conditions for the associated regions would have deviated from the average value because of the meteorological characteristics in mountainous regions. Such variations could increase the crash risk due to winter-related risk factors, such as black ice, affecting the injury severity. The other interpretation is based on “spring fever,” which implies distraction, restlessness, and excitement associated with the beginning of spring. Although there is no official medical diagnosis for this, the change in season could affect a person’s mood, which could result in more distracted driving and contribute to severe injury crashes.

It is found that crashes that occur under sunny weather conditions outside the tunnel increase the likelihood of severe injuries. This is consistent with a previous study [2], which indicated that the likelihood of severe injuries in tunnel crashes appears to be higher in clear weather conditions, compared to adverse weather conditions (i.e., fog, rain, sleet/snow, and severe wind). The result can be interpreted from two perspectives: driving behavior and the visual adaptation of drivers. First, adverse weather conditions such as rain and snow could reduce the friction between the tires and the road surface. Therefore, drivers tend to be more cautious in their driving behaviors, such as reducing speed and paying more attention [2,24,46,82], and such behaviors could contribute to reducing injury severity in tunnel crashes. The other interpretation can be made based on the visual adaptation of drivers in a dark tunnel environment. Although roadway management and operation agencies such as KEC control the tunnel lighting to minimize visual problems such as the black hole and white hole effects, the driver’s eye adaptation problem is inevitable due to the fast transition from the high outside luminance to the low luminance, specifically during the day [83,84]. This eye adaptation problem is more severe in sunny weather conditions than in adverse weather conditions, including snowy, rainy, foggy, and cloudy conditions.

In the study, the major portion of crashes occurred on dry road surface conditions (83.7%). Other surface conditions include wet roads, snow, and ice produced by vehicles carrying rain and snow. That means other surface conditions imply adverse weather conditions, such as rain and snow. As described, drivers tend to be more cautious in their driving behaviors, such as speed and attention, under wet and snow surface conditions [2,24,46,82]. Therefore, the dry tunnel surface condition could have a positive effect on the injury severity factor. This result confirms a similar study by Ma, Chien, Dong, Hu and Xu [2], which showed that crashes under adverse weather conditions are less severe than those under normal weather conditions.

#### 5.3.3. Tunnel and Crash Factor

Three tunnel environment variables were statistically significant: Interior_zone, Tunnel_length, and tunnel_width. However, these variables regarding the tunnel factor did not directly affect the injury severity but did affect the crash factor, and consequently, the crash factor was found to affect the injury severity factor. According to previous studies [1,3,5,21,23,26], it is found that the tunnel factor positively affects severe injuries. Although the results of the previous studies and this study show similarity in the relationship between the tunnel factor and the injury severity factor, this study explains the relationship between these two factors through the crash factor. Therefore, the crash factor in this study indirectly affects injury severity in tunnel crashes. Specifically, crashes that occurred in interior zones, in longer tunnels, and wider tunnels are associated with rear-end collisions and secondary collisions with other vehicles, and consequently, such types of crashes are more likely to result in severe injuries compared to other types of collisions.

When considering drivers’ behavior in tunnels, driving along a dark and narrow tunnel environment may cause anxiety, uncertainty, and even a fear of hitting another vehicle or tunnel walls, as well as other dangerous situations, such as fires and tunnel collapse [6,21]. Therefore, drivers tend to reduce their speed and increase their vigilance. On the other hand, as the driving time and distance in tunnels increase, drivers can adapt to the poor tunnel environment. The low illumination and monotonous and closed environment of tunnels could make drivers more likely to lose their sense of speed, which could easily result in speeding [85]. Moreover, drivers can reduce driving attention and vigilance for longer and wider tunnels. Then, the stopping distance could increase in emergency situations, which could increase rear-end collisions and secondary collisions. However, although drivers tend to increase their speed at the exit zone to leave the tunnel quickly, they could increase their driving attention and vigilance to overcome the “white hole” effect. Pervez, et al. [86] indicated that crashes caused by speeding occurred least frequently in the exit zone in case the tunnel zone was divided into four groups.

Although this study did not apply a variable regarding crash speeds, the injury-severity effects in longer and wider tunnels can be explained by speeding effects (i.e., higher crash speed) caused by reduced driving attention and vigilance. Caliendo et al. [21], Ma et al. [2], and Zhou et al. [26] also found that a greater tunnel length leads to a higher probability of severe injuries. Moreover, wider roads are presumed to decrease the perceived risk, so that drivers might increase their risky behavior [87]. Alternatively, as road width increases, drivers’ speeds tend to increase [88]. Therefore, although the tunnel length and width and the injury severity factor are indirectly associated, their results were confirmed with previous studies.

When considering tunnel environments such as the narrow space and dark conditions, the relationship between the tunnel factor and secondary collision is very natural. Specifically, tunnel crashes that lead to secondary collisions with other vehicles were more likely to have severe injuries, while those that ended in initial collisions or secondary collisions involving a single vehicle tended to have opposite injury patterns. This may be due to the impact energy. Because a secondary collision is defined as two or more impacts, its impact energy should be greater than that of the initial collision alone. Moreover, a comparison of two types of secondary collisions showed that the multi-vehicle type was more severe than the other type. Additionally, although only limited studies have been conducted with crashes that occurred in the roadway environment rather than in the tunnel environment, they concluded that secondary collisions tend to have a higher probability of severe injuries than when there are no secondary collisions [9,10,11,12,13,15,16,56]. Therefore, the result of this study is consistent with previous studies.

Since lane changing is restricted in most sections of freeway tunnels in the Korean roadway system, it is not surprising that the tunnel factor positively affects rear-end collisions. In this study, the collision types were categorized into rear-end and other collisions. Other types of collisions include collisions between a vehicle and tunnel facilities, such as a tunnel wall, as well as collisions between a vehicle and other objects, such as debris and falling objects from other vehicles or an inner wall. Therefore, unlike vehicle-to-object collisions, vehicle-to-vehicle collisions (e.g., rear-end collisions) could result in more severe injuries, which is consistent with the findings in previous studies [26,27]. Another interpretation can be made using the crash angle. Because the right and left sides of tunnels are walls, the initial collisions between a vehicle and the tunnel wall tend to be small-angle lateral crashes due to the narrow lateral clearance. As a result, this type of collision could have less severe injuries.

## 6. Conclusions

This study examined factors affecting the injury severity in crashes with the consideration of secondary collisions in Korean freeway tunnels. Unlike previous studies, we investigated complex relationships between multiple exogenous variables and endogenous variables by considering the direct and indirect relationships between them, using an SEM. As a result, this study showed that the driver, environment, and crash characteristics directly affect injury severity in tunnel crashes. On the other hand, tunnel environments indirectly affect injury severity through the crash characteristics, including rear-end collisions and secondary collisions with other vehicles, rather than directly. Moreover, we found that crashes involving drivers younger than 40 years old decrease the likelihood of severe injury. By contrast, ten variables increased the likelihood of severe injuries, which involved hospitalization for more than three weeks, and fatalities: crashes by male drivers, crashes by trucks, crashes in March, crashes under sunny weather conditions, crashes on dry surface conditions, crashes in interior zones, crashes in wider tunnels, crashes in longer tunnels, rear-end collisions, and secondary collisions with other vehicles.

This study can be differentiated from previous studies because accurate data on secondary collisions were reconstructed using CCTV cameras. Furthermore, based on the high-quality crash data, this study proved that secondary collisions with other vehicles increase injury severity in tunnels. Moreover, older age groups in traffic accident analysis have been conventionally defined as those aged 65 years or older. Although more detailed research is required for generalization, this study found that the injury severity in tunnel crashes according to the driver age group might be distinct at 40 years of age. Lastly, injury severity can be expressed as the number of fatalities, the number of injured, and the level of injury severity. Most studies on the injury severity of tunnel crashes have employed one of these severity variables. On the other hand, this study used an SEM to identify the complex relationship among these severity variables and handle the complex relationships among endogenous and exogenous variables simultaneously.

These findings could form a basis for developing plans and technologies to reduce the severity of crash injuries in freeway tunnel environments. Specifically, policies and strategies to reduce rear-end collisions and secondary collisions with other vehicles will help to reduce injury severities in tunnel crashes. For example, guidelines could be developed for designing tunnels or installing various tunnel facilities, such as improved lighting to reduce temporary ocular blindness in tunnels, technological approaches to warn against the loss of speed sense due to the low illumination and monotonous environment, operational strategies for incident rescue teams to reduce injury severities for longer tunnels, and operational plans for variable message signs in tunnels to reduce secondary collisions by providing crash information in advance. Additionally, simple traffic control devices for driver attention guidance and speed reduction, such as warning signs, perceptual treatments, and delineation treatments, can also help to positively reduce injury severities and secondary collisions [89]. Moreover, as noted by Rella Riccardi, et al. [90], educational campaigns can be an excellent tool to motivate drivers to undertake safety-oriented behavior in freeway tunnels. In addition, advanced road and vehicle technologies such as advanced driving assistant systems (ADASs) and intelligent transportation systems (ITS) will help to reduce secondary collisions in tunnel crashes, and they will, in turn, reduce injury severities.

On the other hand, the relationship between secondary collisions and injury severity has not been fully explored. Because the secondary collision data used in this study were obtained using high-definition CCTV cameras, future studies should investigate the relationship more deeply. Two injury categories (i.e., an incapacitating injury requiring hospitalization for more than three weeks and a fatal injury) were combined into KSI owing to their low frequency. In addition, the independent variable regarding March in the estimated model also had low frequency, accounting for only 12 cases out of 1790. Therefore, further study will be needed to verify the effects of this variable on the injury severity with more tunnel crash data.

## Figures and Tables

**Table 1:** Summary of studies analyzing the injury severity of tunnel crashes.

Authors	Country	Crash Data	Methodology	Key Findings
Amundsen and Ranes [1]	Norway	Police-reported data (1992–1996, 587 road tunnels, and 499 injury crashes)	Descriptive statistics	- The proportion of severe injuries in tunnel crashes is higher than that for open road stretches. - Most fatalities were found in crashes in the mid-zone (150 m inside the tunnel or more).
Ma, Shao, and Zhang [3]	Shaoguan, China	Police-reported data (2003–2004, 4 freeway tunnels, 134 crashes)	Descriptive statistics	- Injury severity in a freeway tunnel is usually higher than those on open freeway stretches. - Injury severities in the mid-zone (300 m inside the tunnel or more) are significantly higher than those in other zones.
Amundsen and Engebretsen [4]	Norway	Police-reported data (2001–2006, 797 national road tunnels, and 926 injury crashes)	Descriptive statistics	- The fatality numbers per crash increase with increasing distance from the tunnel opening. - Crashes that occur in tunnels exceeding 500 m are more severe than those in shorter tunnels.
Caliendo and De Guglielmo [20]	Italy	Motorway Management Agencies (MMA) (2006–2009, 195 Italian motorway tunnels, and 762 crashes)	Descriptive statistics	- Severe crash rate in tunnels was higher than those in open stretches.
Caliendo, et al. [21]	Italy	Motorway Management Agencies (MMA) (2006–2009, 260 Italian motorway tunnels, and 2304 crashes)	Bivariate negative binomial model	- The number of both non-severe and severe crashes in tunnels increased with increasing tunnel length, annual average daily traffic per lane, the percentage of trucks, and the number of lanes.
Yeung and Wong [5]	Singapore	Incident data by Land Transport Authority (LTA) and police-reported data (2009–2011, 3 Singapore expressway tunnels, and 608 crashes)	Descriptive statistics	- Crashes in the interior zones of tunnels were more likely to result in severe injury than those in transition zones (first 250 m inside and outside the tunnel).
Lu, et al. [22]	Shanghai, China	Police-reported data (13 Shanghai river-crossing tunnels, 167 injury crashes)	Descriptive statistics	- Most injury-related crashes occurred in the first 50 m in front of the tunnel openings and the mid-zone (150 m or more inside the tunnel).
Lu, et al. [23]	Shanghai, China	Police-reported data (2011–2012, 14 Shanghai river-crossing tunnels, and 4539 crashes)	Ordered logit model (OLM)	- Crashes in the interior zones of tunnels were more likely to result in severe injury than those in transition zones (first 100 m inside and outside the tunnel). - A higher number of lanes in a tunnel tended to increase the likelihood of severe injuries. - ;Longer tunnels contributed to higher injury severity. - Other variables found to have a more significant effect on injury severity in tunnel crashes: involvement of a heavy-duty truck, 3+ vehicles involved, midnight crashes, wet road surface, male drivers, and older (65+) drivers.
Ma, Chien, Dong, Hu and Xu [2]	China	Police-reported data (2003–2004, 4 freeway tunnels, and 134 crashes)	Generalized ordered logit model	- Crashes that occurred in transition zones (first 100 m inside and outside the tunnel) contributed to higher injury severities than those that occurred in interior zones. - Longer tunnels were associated with higher injury severities. - Crashes that occurred under good weather conditions tended to have higher injury severity than those under severe weather conditions. - Crashes that occurred during the daytime had higher injury severities than those during night-time.
Huang, et al. [24]	Hunan Province, China	Police-reported data (October 2011–September 2016, 12 freeway tunnels, and 1537 crashes)	Classification and regression tree (CART)	- The most critical determinant for injury severity was driving behavior, followed by crash time, grade, curve radius, and vehicle type.
Chen, et al. [25]	Shanghai, China	Shanghai Transport and Port Authority (2014–2016, 6 river crossing tunnels, and 1813 truck crashes)	Ordered logit model (OLM)	- Drivers who were male, aged (65+), and fatigued had a more significant effect on the injury severity in truck-involved tunnel crashes. - The greater the number of lanes, the lower the risk of severe injury in crashes. - Crashes in the mid-zone (100 m inside the tunnel or more) contributed to higher injury severity than those in transition zones. - Tunnel length was positively associated with higher injury severity.
Zhou, et al. [26]	Guizhou Province, China	Police-reported data (2018, freeway tunnels, and 591 crashes)	Two-level binary logistic model	- Tunnel length, truck involvement, rear-end crash, and sequential crash contributed to higher injury severity in tunnels.
Jung and Qin [27]	Korea	Korea Expressway Cooperation (2013–2016, Korean freeway tunnels, and 1564 crashes)	Random forest algorithm	- Adverse weather, fatigued and distracted drivers, tunnel exits, wide tunnels, smaller curve radius (<1800 m), and heavy vehicles were found to be associated with serious injury crashes.

**Table 2:** Definitions and descriptive statistics of the candidate variables.

Group	Category	Description of Observed Variables	Injury Severity		
			PDO	Minor Injury	KSI
Injury severity group	Injury severity	Injury severity (1 if PDO, 2 if minor injury, and 3 if KSI)	1542	180	68
		Number of injured	mean = 0.20; std = 0.78; min = 0; max = 16		
		Number of fatalities	mean = 0.03; std = 0.21; min = 0; max = 4		
Driver group	Driver characteristics	Age_group_1 (1 if younger than 40 years old, 0 otherwise)	895	107	24
		Age_group_2 (1 if aged 40 to 64 years old, 0 otherwise)	592	67	39
		Age_group_3 (1 if aged 65 years old or more, 0 otherwise)	55	6	5
		Gender (1 if male driver, 0 otherwise)	1322 (220)	159 (21)	65 (3)
	Vehicle types	Sedan (1 if sedan or sports utility vehicle, 0 otherwise)	1025	100	29
		Bus (1 if bus, 0 otherwise)	81	17	6
		Truck (1 if truck, 0 otherwise)	376	55	30
		Others (1 if tow truck or military vehicle, 0 otherwise)	60	8	3
Crash property group	Crash characteristics	Vehicle_defects (1 if crash due to vehicle defects, 0 otherwise)	166	10	1
		Speeding (1 if crash due to speeding, 0 otherwise)	244	15	4
		Safety_distance (1 if crash due to following too closely, 0 otherwise)	165	30	9
		Drowsy (1 if crash due to drowsy driving, 0 otherwise)	263	51	30
		Distraction (1 if crash due to distraction, 0 otherwise)	416	67	24
		DUI (1 if crash due to drunk driving, 0 otherwise)	35	3	0
		Debris (1 if crash due to road debris, 0 otherwise)	192	3	0
		Road_damage (1 if crash caused by road damage, 0 otherwise)	61	1	0
		Rear_end (1 if rear-end collision, 0 otherwise)	440 (1102)	111 (69)	48 (20)
	Secondary collision	Initial_collision (1 if initial collision only, 0 otherwise)	1054	75	31
		Secondary_collision_single (1 if secondary collision by single-vehicle alone, 0 otherwise)	164	18	3
		Secondary_collision_multi (1 if secondary collision with other vehicles, 0 otherwise)	324	87	34
Tunnel group	Tunnel characteristics	Entrance_zone (1 if crash occurred in the first 100 m inside the tunnel entrance, 0 otherwise)	195	21	11
		Interior_zone (1 if the crash occurred in the tunnel interior zone, 0 otherwise)	1189	128	48
		Exit_zone (1 if crash occurred in the first 100 m inside the tunnel exit, 0 otherwise)	158	31	9
		Tunnel_length (scaled in km)	mean = 1.2; std = 1.07; min = 0.05; max = 10.97		
		Tunnel_width (scaled in m)	mean = 11.97; std = 1.93; min = 7.2; max = 20.4		
		Tunnel_height (scaled in m)	mean = 7.43; std = 0.74; min = 3.5; max = 10.4		
		Number_lane (1 if the number of directional lanes is 3 or more, 0 otherwise)	224 (1318)	29 (151)	11 (57)
		Curve (1 if horizontally curved tunnel, 0 if horizontally straight tunnel)	107 (1435)	9 (171)	5 (63)
		Grade (1 if vertically curved tunnel, 0 otherwise)	183 (1359)	25 (155)	12 (56)
Environmental group	Environmental characteristics	Snowy (1 if weather conditions outside the tunnel were snowy, 0 otherwise)	60	3	0
		Sunny (1 if weather conditions outside the tunnel were sunny, 0 otherwise)	1017	133	56
		Rainy (1 if weather conditions outside the tunnel were rainy, 0 otherwise)	245	16	6
		Foggy (1 if weather conditions outside the tunnel were foggy, 0 otherwise)	3	1	0
		Cloudy (1 if weather conditions outside the tunnel were cloudy, 0 otherwise)	217	27	6
		Dry (1 if the road surface inside the tunnel was dry, 0 otherwise)	1270 (272)	165 (15)	63 (5)
		January (1 if January, 0 otherwise)	105	11	5
		February (1 if February, 0 otherwise)	116	16	5
		March (1 if March, 0 otherwise)	108	13	12
		April (1 if April, 0 otherwise)	126	15	3
		May (1 if May, 0 otherwise)	143	19	10
		June (1 if June, 0 otherwise)	122	15	5
		July (1 if July, 0 otherwise)	160	16	1
		August (1 if August, 0 otherwise)	154	21	6
		September (1 if September, 0 otherwise)	140	10	6
		October (1 if October, 0 otherwise)	136	18	5
		November (1 if November, 0 otherwise)	119	14	4
		December (1 if December, 0 otherwise)	113	12	6
		Nighttime (1 if the crash occurred from 18:30 to 06:29, 0 otherwise)	453 (1089)	44 (136)	22 (46)

Note 1: The values in parentheses imply the associative frequency of a 0-coded variable in the dichotomous variables. Note 2: In this study, the driver indicates the driver of the vehicle that caused the crash. Additionally, since only one characteristic regarding vehicle types was available, it was included in the driver group as reported by Lee et al. [48].

**Table 3:** SEM terms and notation.

Term	Notation	Dimension
Endogenous latent variable	η	m×1 vector
Exogenous latent variable	ξ	k×1 vector
Causal relationship (exogenous variable → endogenous variable)	Γ	m×k matrix of γ
Causal relationship (endogenous variable → endogenous variable)	B	m×m matrix of β
Endogenous variable error	ζ	m×1 vector
Exogenous variable	x	q×1 vector
Endogenous variable	y	p×1 vector
Indicator loading	$\Lambda_{x}$	q×k $\;\mathrm{matrix}\;\mathrm{of}\;\lambda_{x}\;$
Indicator loading	$\Lambda_{y}$	p×m $\;\mathrm{matrix}\;\mathrm{of}\;\lambda_{y}\;$
Errors for observed variable x	δ	q×1 vector
Errors for observed variable y	ε	p×1 vector

**Table 4:** Recommendations for some rule-of-thumb criteria for goodness-of-fit indices.

Fit Measure	Good Fit	Acceptable Fit
$\chi^{2}/df$	≤2	<5
CFI	0.97≤CFI≤1.00	0.95≤CFI≤0.97
CFI	0.95≤CFI≤1.00	0.90≤CFI≤0.95
AGFI	0.90≤AGFI≤1.00	0.85≤AGFI≤0.90
RMSEA	0.00≤RMSEA≤0.05	0.05≤RMSEA≤0.08

Note: df indicates the degree of freedom.

**Table 5:** Fit measures for model assessment.

Fit Measure	Good Fit	Acceptable Fit	Estimated Model
$\chi^{2}/df$	≤2	$\chi^{2}/df<5$	221.302/68 = 3.254 (acceptable)
RMSEA	0≤RMSEA≤0.05	0.05<RMSEA≤0.08	0.036 (good)
CFI	0.97≤CFI≤1.00	0.95≤CFI<0.97	0.922 (-)
GFI	0.95≤GFI≤1.00	0.90≤GFI<0.95	0.996 (good)
AGFI	0.90≤AGFI≤1.00	0.85≤AGFI<0.90	0.995 (good)

## Data Availability

Data are unavailable because the authors do not have permission to redistribute the data.
